# Disruption of clock gene expression in human colorectal liver metastases

**DOI:** 10.1007/s13277-016-5231-7

**Published:** 2016-08-04

**Authors:** Sander A. Huisman, Ali R. Ahmadi, Jan N. M. IJzermans, Cees Verhoef, Gijsbertus T. J. van der Horst, Ron W. F. de Bruin

**Affiliations:** 1 Department of Surgery, Erasmus University Medical Center, Wytemaweg 80, 3015 CN Rotterdam, Netherlands; 2 Department of Molecular Genetics, Erasmus University Medical Center, Wytemaweg 80, 3015 CN Rotterdam, Netherlands

**Keywords:** Circadian clock, Rhythms, Colorectal liver metastases, Colorectal cancer

## Abstract

The circadian timing system controls about 40 % of the transcriptome and is important in the regulation of a wide variety of biological processes including metabolic and proliferative functions. Disruption of the circadian clock could have significant effect on human health and has an important role in the development of cancer. Here, we compared the expression levels of core clock genes in primary colorectal cancer (CRC), colorectal liver metastases (CRLM), and liver tissue within the same patient. Surgical specimens of 15 untreated patients with primary CRC and metachronous CRLM were studied. Quantitative real-time polymerase chain reaction (qRT-PCR) was used to measure the expression of 10 clock genes: *CLOCK*, *BMAL1*, *PER1*, *PER2*, *PER3*, *CRY1*, *CRY2*, *CSNK1E*, *TIM*, *TIPIN*, and 2 clock-controlled genes: *Cyclin-D1*, and *WEE1*. Expression levels of 7 core clock genes were downregulated in CRLM: *CLOCK* (*p* = 0.006), *BMAL1* (*p* = 0.003), *PER1* (*p* = 0.003), *PER2* (*p* = 0.002), *PER3* (*p* < 0.001), *CRY1* (*p* = 0.002), and *CRY2* (*p* < 0.001). In CRC, 5 genes were downregulated: *BMAL1* (*p* = 0.02), *PER1* (*p* = 0.004), *PER2* (*p* = 0.008), *PER3* (*p* < 0.001), and *CRY2* (*p* < 0.001). *CSNK1E* was upregulated in CRC (*p* = 0.02). *Cyclin-D1* and *WEE1* were both downregulated in CRLM and CRC. Related to clinicopathological factors, a significant correlation was found between low expression of *CRY1* and female gender, and low *PER3* expression and the number of CRLM. Our data demonstrate that the core clock is disrupted in CRLM and CRC tissue from the same patient. This disruption may be linked to altered cell-cycle dynamics and carcinogenesis.

## Introduction

The circadian timing system controls important biological processes, including metabolic and proliferative functions [1–3]. The rhythmic behavior of these processes takes approximately 24 h and is called circadian rhythm (rhythms of approximately 1 day). The circadian clock consists of a master oscillator which is located in the neurons of the suprachiasmatic nuclei (SCN) in the anterior hypothalamus of the brain [4–6]. The master circadian oscillator coordinates peripheral circadian clocks through both the autonomic nervous system and neuroendocrine systems in most cells of the body [7]. Peripheral circadian oscillators all consist of the same set of clock genes but regulate their expression in a tissue-specific way [8].

The human molecular clock system involves a set of core clock genes that act in transcription-translation feedback loops. The primary feedback loop consists of CLOCK (circadian locomotor output cycles kaput) and BMAL1 (brain-muscle Arnt-like protein 1) which heterodimerize and subsequently activate transcription of the *Cryptochrome* (*CRY1* and *CRY2*) and *Period* (*PER1*, *PER2*, and, *PER3*) genes by binding to E-box elements in their promoters. The PER and CRY proteins translocate to the cytoplasm where PER proteins are phosphorylated by CKIɛ. Phosphorylated PER proteins are unstable and are degraded by ubiquitination. CRY proteins promote the formation of PER/CRY complexes and re-enter the nucleus, where they inhibit the transcription of their own genes by blocking CLOCK/BMAL1. This molecular core oscillator is coupled to circadian output processes through a series of clock-controlled genes (CCGs), which together regulate about 40 % of the transcriptome [9–11].

Perturbations in the function of circadian clock genes may have significant effects on human health, and may cause sleep disorders, depression, and gastrointestinal and cardiovascular diseases. Furthermore, the circadian timing system plays an important role in the development of cancer. Epidemiological studies have demonstrated that circadian disruption in shift workers increases the risk of various epithelial cancers [12–15]. An important part of the cell cycle is regulated by the circadian clock. CLOCK/BMAL1 directly regulates cell-cycle genes that control cell proliferation, DNA damage, and apoptosis. These CCGs include *WEE-1* and *Cyclin-D1*. Disruption of the circadian timing system may lead to a deregulated cell cycle which favors carcinogenesis [16].

It has been demonstrated that inhibition of *Per1* caused reduced apoptosis in HCT116 colon cancer cells, while overexpression of *Per1* leads to DNA damage-induced apoptosis [17]. Inactivation of *Per2* caused deregulation of *Bmal1* expression which contributed to a high incidence of tumor formation. In addition, mice deficient in *Per2* showed an increase in tumor formation after ɣ-radiation [18]. Recently, we showed that the core clock machinery is severely disrupted in murine colorectal liver metastases (CRLM) and that the presence of tumor in the liver induces a phase shift in the liver and kidney tissue clocks [19].

In humans, CRLM worsen the prognosis of almost 60 % of patients with colorectal cancer [20]. In animal models, the core clock machinery is disrupted in several types of cancer. The functioning of the circadian clock in patients with CRLM has remained unclear. A better understanding of how tumors affect the circadian clock may help elucidate the role of the clock in cancer patients. We therefore investigated the expression levels of core clock genes in human CRLM tissue, adjacent liver tissue, and the primary colorectal tumor. Furthermore, we related the expression levels to clinicopathological factors in these patients.

## Material and methods

### Patients

Surgical resection specimens of the primary colorectal tumor, liver metastases, and adjacent normal liver tissue were obtained from 15 CRLM (male: 8, female: 7) patients who did not receive neo-adjuvant chemotherapy treatment. The patients underwent surgery at the Erasmus MC Cancer Institute, Erasmus University Medical Center, Rotterdam, The Netherlands between January 2005 and January 2012. Clinical data including tumor characteristics of these patients are shown in Table 1. All operations started between 8:00 a.m. and 11:00 a.m. and the average time patients were in the operation room was 3.30 h. All tissues were collected between 09:00 a.m. and 13:30 p.m. and immediately frozen into liquid nitrogen and stored at −80 °C until further analysis. Informed consent was obtained from all patients and the study was approved by the Ethics Committee at our institution.

**Table 1:** Characteristics of clinicopathological factors from 15 patients evaluated for circadian rhythm and outcome

	*N* (%)	Male/female
Age (years), mean (±SD)	67.5 ± 9.8	M 66.9 ± 10.2
		F 68.3 ± 10.0
Sex		
Male (M)/female (F)		8/7
Number of metastases		
1	7 (46.7)	M4/F3
2	3 (20.0)	M2/F1
3	2 (13.3)	M1/F1
4	1 (6.7)	M0/F1
5	1 (6.7)	M0/F1
6	1 (6.7)	M1/F0
Diameter of largest metastasis (cm)		
1.20–2.20	5 (33.3)	M2/F3
2.40–4.40	7 (46.7)	M4/F3
5.00–9.00	3 (20.0)	M2/F1
CRLM in number of segments		
1	4 (26.7)	M3/F1
2	6 (40.0)	M3/F3
3	1 (6.7)	M0/F1
4	4 (26.7)	M2/F2
Primary tumor location		
Ascending colon	2 (13.3)	M1/F1
Transverse colon	–	–
Descending colon	5 (33.3)	M4/F1
Ascending+descending colon	1 (6.7)	M0/F1
Rectum	7 (46.7)	M3/F4
Histological type CRC		
Moderately differentiated adenocarcinoma	13 (86.7)	M7/F6
Poorly differentiated adenocarcinoma	2 (13.3)	M1/F1
Depth of tumor invasion CRC		
T1	–	–
T2	4 (26.7)	M2/F2
T3	9 (60.0)	M5/F4
T4	1 (6.7)	M1/F0
Missing	1 (6.7)	M0/F1
Lymph node metastasis CRC		
N0	8 (53.3)	M5/F3
N1–N2	7 (46.7)	M3/F4

### Sampling procedure

All surgical resection specimens from primary colorectal tumor, liver metastases, and adjacent normal liver tissue were first macroscopically, then microscopically identified by an experienced pathologist. Frozen resection specimens were retrieved from the archives of the pathology department and at least 1 cm in diameter of viable tumor tissue was included using a frozen tissue slicer (Leica CM1850 UV, Leica Biosystems). There was no admixture of stromal tissue and no necrosis was identified in the included samples. Tumor characteristics are shown in Table 1.

### Fresh frozen tissue, RNA extraction, and cDNA synthesis

RNA was isolated from all tissues by phenol extraction using Trizol reagent (Invitrogen, Carlsbad, CA, USA) according to the manufacturer’s instructions. The amount of extracted RNA was measured by Nanodrop Spectrophotometry (Nanodrop Technology, Wilmington, DE, USA). To avoid genomic DNA contamination, RNA was purified by DNAse treatment (RQ1 RNase-Free DNase; Promega, Madison, WI, USA). RNA was then reverse transcribed into complementary DNA (cDNA) using random primers (Invitrogen) and Superscript II RT (Invitrogen). cDNA samples were stored at −20 °C until further analysis.

### Quantitative real-time reverse transcriptase polymerase chain reaction

Gene expression was analyzed by quantitative real-time PCR (qRT-PCR) to assess differential expression of clock genes in CRC tissue, CRLM, and adjacent liver tissue using an Applied Biosystems 7700 PCR machine (Foster City, CA, USA). RT-PCR was performed using SYBR Green-based Quantitect Primer Assay (Qiagen, Venlo, The Netherlands) for 10 clock transcripts: *CLOCK* (QT00054481), *BMAL1* (QT00011844), *PER1* (QT00069265), *PER2* (QT00011207), *PER3* (QT00097713), *CRY1* (QT00025067), *CRY2* (QT00094920), *TIM* (QT00019789), *TIPIN* (QT00054334), *CSNK1E* (QT02323916), and 2 clock-controlled genes: *Cyclin-D1* (QT00495285), *WEE1* (QT00038199). PCR reactions were carried out in a total volume of 25 *μ*L using the Quantifast SYBR Green PCR kit (Qiagen, Venlo, The Netherlands). Each sample was tested in triplicate according to the following PCR protocol: 10 min at 50 °C, 5 min at 95 °C, followed by 40 cycles at 95 °C for 10 s, and at 60 °C for 30 s. ΔCt values of the genes of interest were calculated as described by the method of Pfaffl et al. using the glutaraldehyde-3-phosphate dehydrogenase (*GAPDH*; QT00079247) as a housekeeping gene [21]. *GAPDH* is a commonly accepted marker for normalization of qPCR data obtained from human tissues. Triplicate values of GAPDH showed low standard deviations, and a one-way ANOVA analysis between GAPDH values of all tissues showed no significant differences (*p* > 0.05). ΔCt values were normalized to the average ΔCt of the normal liver tissue. The fold change was calculated using the Pfaffl equation, 2^-ΔΔCt^. Results are expressed as median with the interquartile range (IQR). The IQR is the difference between the upper and lower quartiles (IQR = Q_3_−Q_1_).

### Statistical analysis

Gene expression levels of CRLM and CRC were compared with those of adjacent liver tissue and calculated using the Pfaffl equation, 2^-ΔΔCt^. To assess the statistical significance of the up- or downregulation of genes, the Wilcoxon signed-rank rest was used. Correlation analyses between gene expression levels and all nine clinical pathological factors was performed using the Spearman correlation. All analyses were corrected for multiple testing by the Bonferroni method. All statistical tests were two-sided and performed using SPSS 21 for Windows software (Statistical Package for Social Sciences, Chicago, IL). *p* < 0.05 was considered to be significant, unless otherwise mentioned.

## Results

### Clock gene mRNA expression in CRLM compared to adjacent liver

**Fig. 1:**
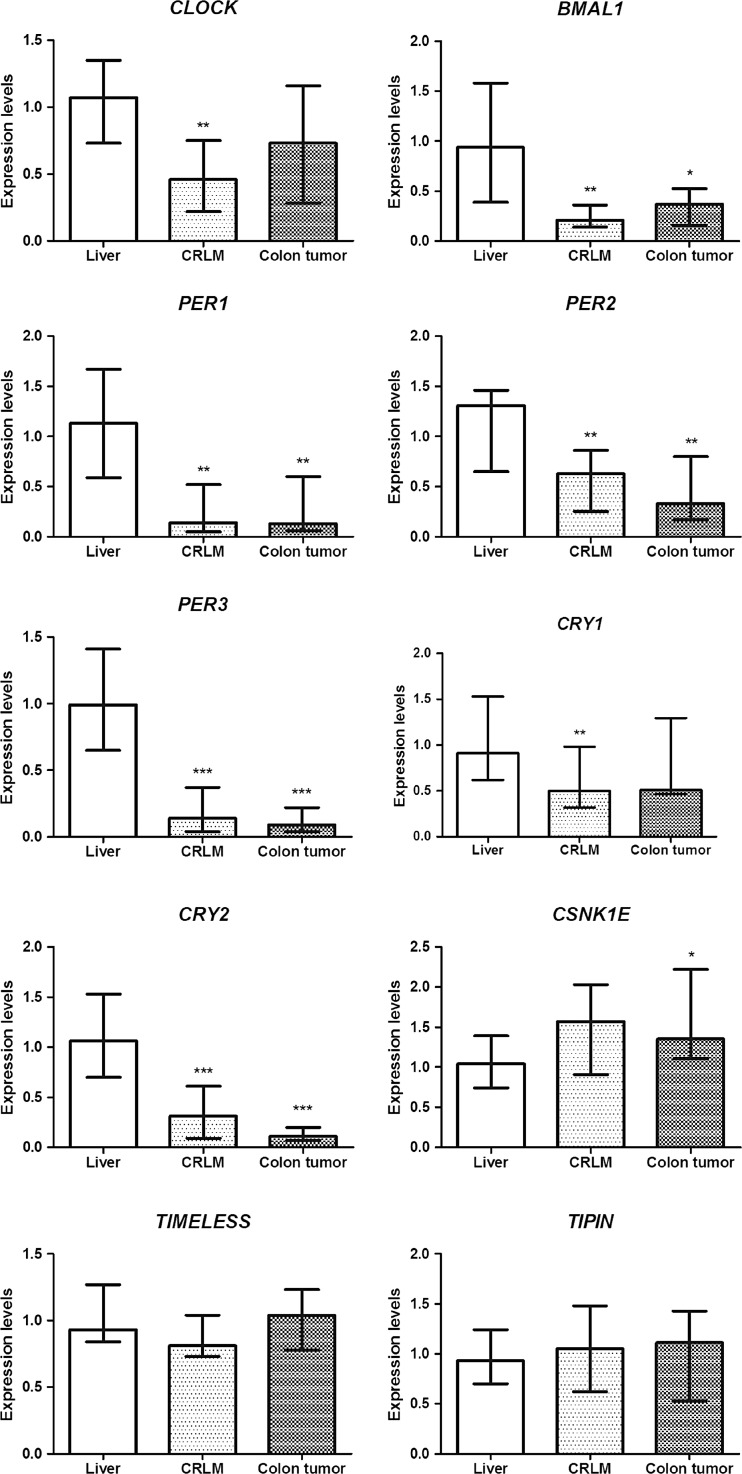
mRNA expression levels of clock and clock-controlled genes in the liver, colorectal liver metastases (CRLM), and colon tumor. The relative mRNA expression of each gene of interest was normalized to glutaraldehyde-3-phosphate dehydrogenase (*GAPDH*). ΔCt values were normalized to the average ΔCt of the normal liver tissue. For each gene, boxes show the median with the interquartile range (IQR = Q3−Q1). *Asterisks* indicate significance of the difference in expression of each gene in the liver as compared to CRLM and CRC as assessed by the Wilcoxon signed-rank test (*p < 0.05, **p < 0.01, ***p < 0.001)

To compare clock gene expression in CRLM with adjacent normal liver tissue, we analyzed mRNA expression levels of 10 clock genes (*CLOCK*, *BMAL1*, *PER1*, *PER2*, *PER3*, *CRY1*, *CRY2*, *CSNK1E*, *TIM*, *TIPIN*). Liver metastasis and liver tissue were used from 15 patients. Relative messenger RNA (mRNA) expression levels of clock genes in the liver and CRLM are presented in Fig. 1. Tumor samples were normalized to the average ΔCt of the liver tissue, and 7 genes were subsequently observed to be downregulated in CRLM: *CLOCK* (median = 0.46, Q1–Q3 = 0.22–0.75, *p* = 0.006), *BMAL1* (median = 0.21, Q1–Q3 = 0.14–0.36, *p* = 0.003), *PER1* (median = 0.14, Q1–Q3 = 0.05–0.52, *p* = 0.003), *PER2* (median = 0.63, Q1–Q3 = 0.25–0.86, *p* = 0.002), *PER3* (median = 0.14, Q1–Q3 = 0.04–0.37, *p* < 0.001), *CRY1* (median = 0.50, Q1–Q3 = 0.32–0.98, *p* = 0.002), and *CRY2* (median = 0.31, Q1–Q3 = 0.09–0.61, *p* < 0.001). The expression levels of TIM and TIPIN showed no significant difference (median = 0.81, Q1–Q3 = 0.73–1.04, *p* = 0.54, and median = 1.05, Q1–Q3 = 0.62–1.48, *p* = 0.74, respectively).

### Clock gene mRNA expression in the primary colon tumor compared to liver tissue

To determine whether clock gene expression was also impaired in the primary tumor, we measured mRNA expression levels of colorectal tumors of the same patients. Five of 10 genes were downregulated, namely *BMAL1* (median = 0.37, Q1–Q3 = 0.16–0.53, *p* = 0.02), *PER1* (median = 0.13, Q1–Q3 = 0.06–0.6, *p* = 0.004), *PER2* (median = 0.33, Q1–Q3 = 0.17–0.80, *p* = 0.008), *PER3* (median = 0.09, Q1–Q3 = 0.04–0.22, *p* < 0.001), *CRY2* (median = 0.11, Q1–Q3 = 0.07–0.20, *p* < 0.001). Again, *CSNK1E* was upregulated (median = 1.35, Q1–Q3 = 1.11–2.22, *p* = 0.02). The expression of four genes did not show significant differences: *CLOCK* (median = 0.73, Q1–Q3 = 0.29–1.16, *p* = 0.43), *CRY1* (median = 0.51, Q1–Q3 = 0.47–1.30, *p* = 0.23), *TIM* (median = 1.04, Q1–Q3 = 0.78–1.23, *p* = 0.52), *TIPIN* (median = 1.11, Q1–Q3 = 0.53–1.43, *p* = 0.70) (Fig. 1).

### Clock-controlled gene mRNA expression in CRLM and CRC compared to liver tissue

**Fig. 2:**
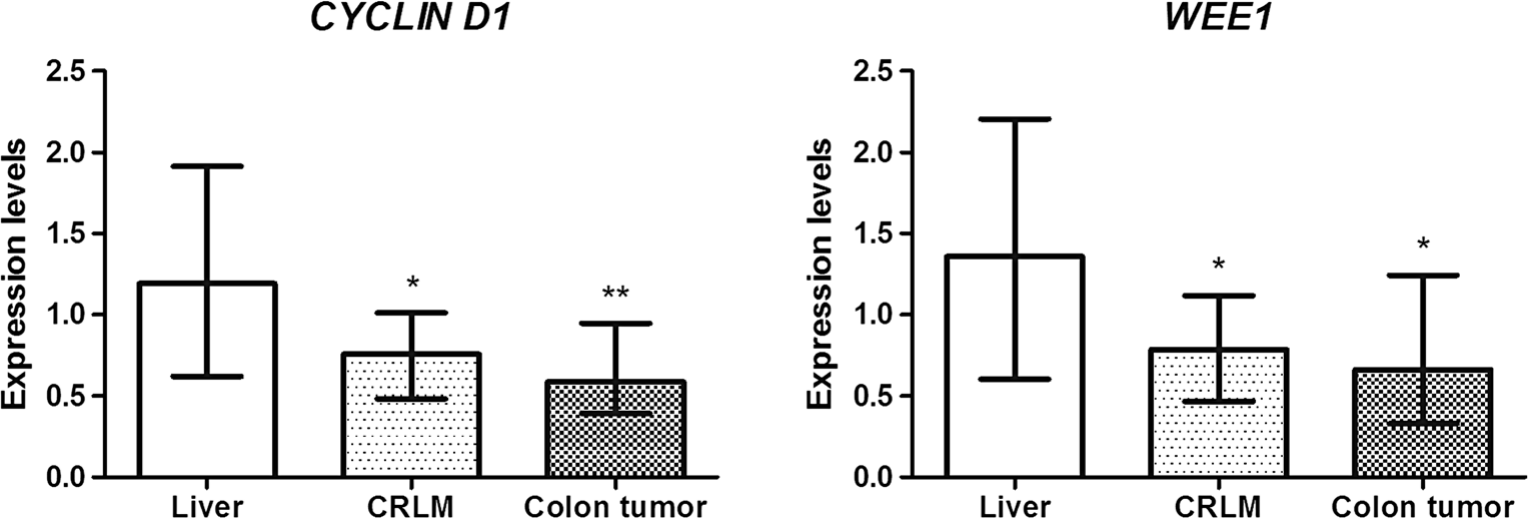
mRNA expression levels of the clock-controlled genes *Cyclin-D1* and *WEE1* in the liver, colorectal liver metastases (CRLM), and colon tumor. The relative mRNA expression of each gene of interest was normalized to glutaraldehyde-3-phosphate dehydrogenase (*GAPDH*). ΔCt values were normalized to the average ΔCt of the normal liver tissue. For each gene, *boxes* show the median with the interquartile range (IQR = Q3−Q1). *Asterisks* indicate significance of the difference in expression of each gene in the liver as compared to CRLM and CRC as assessed by the Wilcoxon signed-rank test (*p < 0.05, **p < 0.01)

To determine whether the expression of clock-controlled genes was impaired in CRLM and the primary tumor, we analyzed mRNA expression of 2 circadian output genes (*Cyclin-D1*, and *WEE-1*). *Cyclin-D1* was downregulated in CRLM (median = 0.76, Q1–Q3 = 0.48–1.01, p = 0.02), and in CRC (median = 0.59, Q1–Q3 = 0.39–0.95, p = 0.008). *WEE-1* was also downregulated in CRLM (median = 0.79, Q1–Q3 = 0.47–1.12, p = 0.04), and in CRC (median = 0.66, Q1–Q3 = 0.33–1.24, p = 0.03) (Fig. 2).

### Relation between clock gene mRNA expression levels and clinicopathological factors

**Fig. 3:**
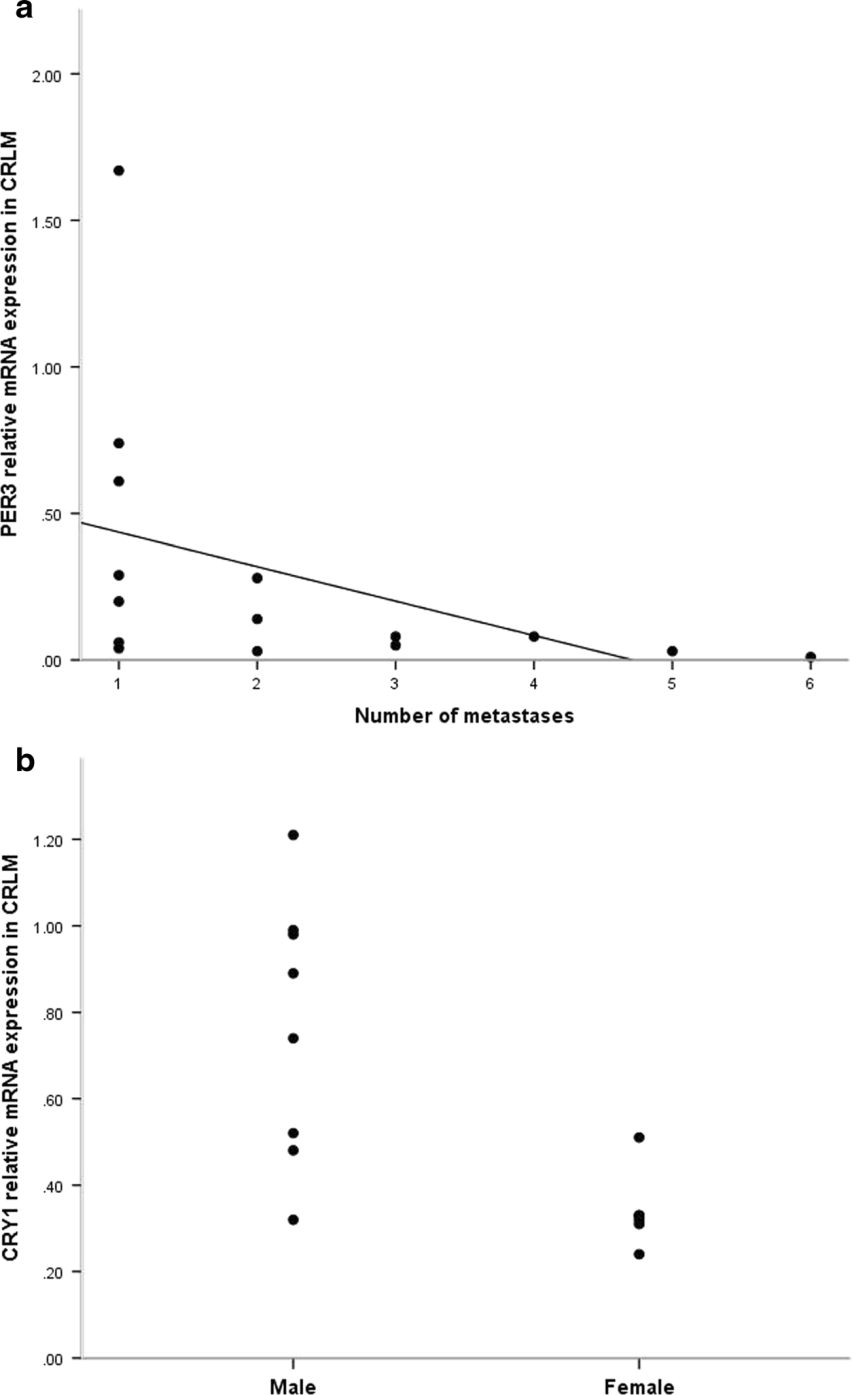
Correlation between clock gene mRNA expression levels, CRLM, and gender. **a** The correlation between *PER3* mRNA expression levels in CRLM and the number of metastases, evaluated by Spearman test (*r* = 0.645, *p* = 0.009). **b** The correlation between *CRY1* mRNA expression levels in CRLM and patient gender evaluated by the Spearman test (*r* = 0.700, *p* = 0.005)

A statistically significant correlation was found between CRLM mRNA levels of *PER3* and the number of metastases. Lower *PER3* mRNA levels were found with an increasing number of metastases (*r* = 0.645, *p* = 0.009) (Fig. 3a). Another significant correlation was found between CRLM *CRY1* mRNA levels and patient gender. Lower *CRY1* mRNA levels were found in female patients compared to male patients (*r* = 0.700, *p* = 0.005) (Fig. 3b). There were no other significant correlations found between mRNA expression levels and clinicopathological factors.

## Discussion

In the current study, we examined the expression levels of clock and clock-controlled genes in colorectal liver metastases (CRLM), the primary colorectal tumor, and liver tissue in surgical resection specimens of CRC patients. We also studied possible relations between gene expression levels and clinical and pathological factors of these patients. We used quantitative real-time polymerase chain reaction (qRT-PCR) to identify the expression levels of *CLOCK*, *BMAL1*, *PER1*, *PER2*, *PER3*, *CRY1*, *CRY2*, *CSKN1E*, *TIM*, *TIPIN*, *Cyclin-D1*, and *WEE1*. We observed a downregulation of core clock, as well as of clock-controlled gene mRNA expression levels in both liver metastases and colorectal cancer. The genes encoding CLOCK and BMAL1, the two core clock proteins that heterodimerize and drive transcription of clock (controlled) genes, were both significantly downregulated in CRLM. *BMAL1* expression was also lowered in colorectal tumors. In line with the lower expression levels of *CLOCK* and *BMAL1*, genes activated by the CLOCK/BMAL1 complex, such as *PER1*, *PER2*, *PER3*, *CRY1*, and *CRY2* all show a significant reduction in expression compared to normal liver tissue. The only gene that was significantly upregulated in the primary tumor was *CSKN1E*. We observed no differences in the expression levels of *TIM* and *TIPIN*.

To our knowledge, this is the first study describing downregulation of clock genes in human CRLM. Our findings are in line with previous studies describing circadian disruption in other malignancies. In more than 95 % of breast cancer tissue from 55 women, expression levels of *PER1*, *PER2*, and *PER3* were severely disrupted in comparison with adjacent non-cancerous tissue [22]. Pancreatic cancer has a low incidence rate, but is very aggressive with high mortality rates. Especially *PER1* and downstream effectors of the circadian clock are lower expressed in pancreatic cancer which further suggests they may act as tumor suppressor genes in healthy tissue [23]. Our data are supported by a study in human primary colorectal cancer. A downregulation in expression of *BMAL1*, *PER1*, *PER2*, *PER3*, and *CRY2* was found. Furthermore, differential expression of clock genes was associated with differences in survival [24]. In a study of 202 untreated CRC patients, *PER1* and *PER3* expression levels were significantly lower compared to normal tissue. In contrast, the expression of *CLOCK* and *CKIɛ* was significantly higher in cancer tissue. *PER2* was shown to be differentially expressed related to survival, with a better survival corresponding with a high *PER2* expression [25].

In this study, the only gene that was significantly upregulated in CRC and showed a trend towards increased expression in CRLM was *CSNK1E*. The *CSNK1E* gene encodes the CKIɛ protein, whose main function is to regulate circadian rhythm by phosphorylation and degradation of Period genes [26]. We showed that *PER1*, *PER2*, and *PER3* gene expression levels were all lower in cancer tissue than in liver tissue, while *CSNK1E* gene expression was higher in cancer tissue. The decreased expression levels of both transcription activator, (BMAL1), and transcription inhibitor genes (CRYs and PERs) suggest that the clock in the primary tumor and CRLM may be dampened and/or less robust. Upregulation of *CSNK1E* may lead to enhanced phosphorylation of the PER2 protein which is known to destabilize the PER protein and target it for ubiquitination and subsequent proteosomal degradation. Furthermore, *CKIɛ* plays an essential role in the early development of CRC. *CKIɛ* is involved in cell proliferation by stabilizing β-catenin and mimicking the effect of WNT-signaling. Subsequently, this will lead to increased levels of β-catenin in the nucleus to control transcription and maintain tumorigenesis [27, 28]. Knocking down *CSNK1E* in a human sarcoma cell line led to growth inhibition of cells, and *CSNK1E* was found to be upregulated in ten different human cancer tissues compared to normal tissue [29].

In contrast to others, we found no significant difference in *TIMELESS* (*TIM*) and timeless-interacting protein (*TIPIN*) mRNA expression levels. These genes interact with components of the DNA replication system to regulate DNA replication processes under normal and stress conditions and are essential in regulating different phases of the cell cycle [30]. Downregulation of *TIM* increased doxorubicin toxicity in HCT116 cancer cells, and it is suggested that *TIM* inhibition could be used to enhance cytotoxic effectiveness of chemotherapeutic drugs [31]. Downregulation of *TIM* and *TIPIN* was found in kidney cancer patients compared to normal kidney tissue [32].

To determine whether the disruption of the clock affects its output in CRLM and CRC, we measured the expression of two CCGs. The mRNA expression of *WEE-1* was downregulated in CRLM as well as in CRC. *WEE-1* is a nuclear kinase which is involved in the regulation of cell-cycle progression, a key regulator of mitoses. The circadian timing system plays an important role in transcription of *WEE-1*. In *Clock-*mutant mice, a low expression of *WEE-1* was found [2]. The mRNA expression of *Cyclin-D1* was also downregulated in CRLM and in CRC. *Cyclin-D1* plays an important role in the cell cycle as well, because it regulates the progression of cells in G1/S transition [33]. The relevance of these perturbations for both tumor biology and as biomarkers for prognosis warrants further study.

We related the expression levels of clock genes to clinical and pathological factors. Low expression of *PER3* was correlated with a higher number of metastases. In another study, low *PER1* expression was correlated with the development of CRLM in CRC patients [25]. We also found a significant correlation between gender and the expression of *CRY1* in CRLM. The lowest levels of *CRY1* mRNA expression were found in female patients. This correlation was also found in a study where differential expression levels of core clock genes were determined in tumor specimens of CRC patients [24]. The fact that female patients show lower *CRY1* expression levels could be related to a difference in metabolic pathways and xenobiotic detoxification between genders. In the Chronotherapy Group Trial, including a schedule of chronomodulated delivery of chemotherapy, female patients were shown to have shorter survival and greater toxicity when treated with 5-fluorouracil and leucovorin [34].

The mRNA expression levels of CRLM of all core clock genes in this study show differential expression compared to liver tissue. These results support the hypothesis of the apparent coupling between the circadian rest-activity cycle and the time-dependent toxicity of drugs, which may be exploited in the field of chronotherapy. The basis of chronotherapy relies on the principle of administering chemotherapy at times when toxicity is expected to be lowest [35, 36]. A phase III study in CRC patients has shown better tolerability and anti-tumor activity compared with conventional therapy when chemotherapy was administered according to the least toxic dosing times [34]. In a phase II study, patients with unresectable CRLM were treated with chronotherapy and highly toxic hepatic arterial infusion which resulted in a doubling of secondary surgical resection rates [37].

Albeit our study shows evidence of a disrupted timing system in CRLM and CRC in patients, we studied a relatively small cohort of patients. By expanding the number of patients, more correlations might be found between mRNA expression levels and clinicopathological factors. The mRNA expression levels of CRC are normalized to the levels of the adjacent liver, but not colon tissue. Based on experiments with rats, it is expected that the circadian timing system in the colon is in phase with that in the liver [38]. Furthermore, we were only able to study gene expression at the timepoint at which the resection specimen was obtained. Since patients are operated on at different times of the day, and surgical resections are not procedures with a fixed time frame, this is a limitation inherent to a clinical study. To further elucidate this issue, we are currently investigating the impact of clock gene expression levels in cancer cells in vitro by knocking down and overexpressing clock genes in various tumor cell lines, followed by systematic phenotyping of cancer properties of the cell (i.e., proliferation rate, cell migration and invasion properties, and drug sensitivity).

In summary, the present study shows that there are differences in clock gene expression in the CRLM and CRC tissue compared to the liver in patients without neo-adjuvant chemotherapy treatment. The differential expression might be related to carcinogenesis, tumor burden, and survival, and supports the application of chronomodulated chemotherapy.
